# *mizer*: an R package for multispecies, trait-based and community size spectrum ecological modelling

**DOI:** 10.1111/2041-210X.12256

**Published:** 2014-09-23

**Authors:** Finlay Scott, Julia L Blanchard, Ken H Andersen

**Affiliations:** 1 Maritime Affairs Unit, IPSC, European Commission Joint Research CentreVia Enrico Fermi 2749, I – 21027, Ispra (VA), Italy; 2 Centre for the Environment Fisheries and Aquaculture Science (CEFAS)Pakefield Road, Lowestoft, NR33 0HT, UK; 3 Department of Animal and Plant Sciences, University of SheffieldAlfred Denny Building, Western Bank, Sheffield, S10 2TN, UK; 4 Centre for Ocean Life, National Institute of Aquatic Resources, Technical University of Denmark2920 Charlottenlund Slot, Charlottenlund, Denmark

**Keywords:** size spectrum, multispecies modelling, trophic level, natural resource management, fisheries

## Abstract

1. Size spectrum ecological models are representations of a community of individuals which grow and change trophic level. A key emergent feature of these models is the size spectrum; the total abundance of all individuals that scales negatively with size. The models we focus on are designed to capture fish community dynamics useful for assessing the community impacts of fishing.
2. We present *mizer*, an R package for implementing dynamic size spectrum ecological models of an entire aquatic community subject to fishing. Multiple fishing gears can be defined and fishing mortality can change through time making it possible to simulate a range of exploitation strategies and management options.
3. *mizer* implements three versions of the size spectrum modelling framework: the community model, where individuals are only characterized by their size; the trait-based model, where individuals are further characterized by their asymptotic size; and the multispecies model where additional trait differences are resolved.
4. A range of plot, community indicator and summary methods are available to inspect the results of the simulations.

## Introduction

Size spectrum ecological models are a way to model a community of individuals that grow and change trophic level during life. The models we focus on are designed to capture fish community dynamics (e.g. Benoît & Rochet 2004; Andersen & Beyer 2006; Andersen *et al*. 2008; Law *et al*. 2009; Hartvig, Andersen & Beyer 2011). They can be used to understand how aquatic communities are organized (Andersen & Beyer 2006; Andersen, Beyer & Lundberg 2009; Blanchard *et al*. 2009) and to simulate the community impact of different fishing scenarios, for example fishing forage fish (Engelhard *et al*. 2014; Houle *et al*. 2013), fishing large fish (Andersen & Pedersen 2010), ‘balanced’ harvesting (Jacobsen, Gislason & Andersen 2014) and recovery after fishing (Andersen & Rice 2010). Size spectrum models can also be used to evaluate ecosystem management strategies (Blanchard *et al*. 2014) and the global economics of fishing (Barange *et al*. 2014). Applications of size spectrum models have focused on marine communities, but they are also applicable for freshwater communities.

We present the R package (R Development Core Team 2014), *mizer*, for size spectrum ecological modelling. The source code for *mizer* is hosted at https://github.com/drfinlayscott/mizer. The package can be installed from CRAN. An extensive vignette is available that includes a full description of the model equations and examples (http://cran.r-project.org/web/packages/mizer/vignettes/mizer_vignette.pdf). The package can be used to simulate the size spectrum dynamics in an aquatic community. It is not a ‘community assessment’ tool (i.e. it does not ‘fit’ the model to assess the current state of the community) but simulates the potential consequences of various fishing patterns, conditional on the model assumptions and parameter values.

## Model description

**Figure 1:**
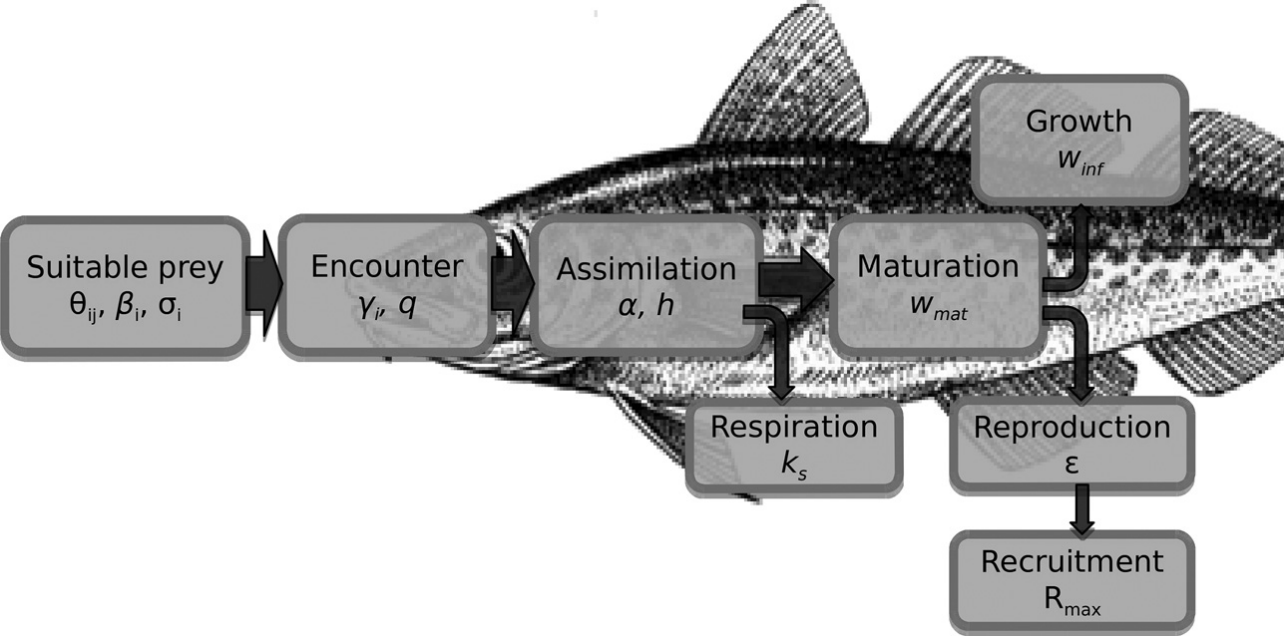
Sketch of the main processes involved in the bioenergetic budget of an individual of species *i* and parameters involved in each process (see the package vignette and Tables S1 and S2). Suitable prey are selected according to the species preference θ_*ij*_ and the size preference parameters. Suitable prey are encountered and assimilated, and used for respiratory costs. The remaining energy is split between growth and reproduction depending on the individual.

Size spectrum models are a subset of physiologically structured models (De Roos & Persson 2001) as growth and maturation are food-dependent, and processes are formulated at the individual level (Fig.1). All parameters in size spectrum models are related to individual size (weight), making it possible to formulate the model with a few general parameters. The model framework builds on two central assumptions. The first is that an individual can be characterized by its weight *w* and its species number *i* only. The model calculates the size and trait spectrum *N*_*i*_(*w*) which is the density of individuals at a particular size. Scaling from individual-level processes of growth and mortality to the size spectrum is achieved by means of the McKendrick–von Foerster equation as follows:


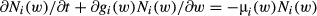
eqn 1

where individual growth *g*_*i*_(*w*) and mortality μ_*i*_(*w*) are determined by the availability of food from other individuals plus a background resource spectrum (modelled dynamically using a semi-chemostat growth equation), predation from other individuals and fishing mortality.

The second assumption is that food preference is determined partly by species preference and partly by individual weight combined with a prey weight preference, described by a log-normal selection model (Ursin 1973) in terms of the ratio between the weight of predators *w* and prey *w*_*p*_


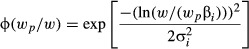
eqn 2

where β_*i*_ is the preferred predator–prey mass ratio and σ_*i*_ the width of the weight selection function. As prey selection is size based, cannibalism is an intrinsic model feature. The rest of the model formulation rests on standard assumptions from ecology and fisheries science about how encounters between predators and prey lead to growth, recruitment and mortality.

Fishing mortality, *F*, is imposed on individuals by fishing gears. *F* is a product of the gear selectivity function (the ability of a gear to capture individuals by size, ranging from 0 to 1), fishing effort (a measure of the fishing intensity) and catchability (an additional scalar that relates population abundance to *F*). Fishing effort can vary through time allowing dynamic harvest patterns to be simulated. Two selectivity functions are included with *mizer*: ‘knife-edge’ (where selectivity increases instantly from 0 to 1 at the specified size) and sigmoid (where selectivity increases with size, similar to a selection pattern from a trawl gear). Users are able to define mass- and length-based selectivity functions and different functions, and parameters can be specified for each species in the model. Full details of the model equations and parameters are in Section 3 of the package vignette.

*Mizer* encompasses three versions of size spectrum models with increasing complexity: the community model (Benoît & Rochet 2004; Maury *et al*. 2007; Blanchard *et al*. 2009; Law *et al*. 2009), the trait-based model (Andersen & Beyer 2006; Andersen & Pedersen 2010; Andersen & Rice 2010) and the multispecies model (Hartvig, Andersen & Beyer 2011; Blanchard *et al*. 2014).

In the community model, individuals are only characterized by their size and represent a single group representing an across-species average. Reproduction is not considered, and the recruitment flux is constant.

The trait-based model resolves a continuum of species with varying asymptotic sizes. The asymptotic size, *W*_*inf*_, is considered to be the most important trait that characterizes the species life history. The continuum is represented by a discrete number of species spread evenly over the range of asymptotic sizes. The number of species is not important and does not affect the general dynamics of the model (Andersen & Pedersen 2010). Many of the parameters, such as β and σ, are the same for all species. Other model parameters are determined by the asymptotic size. For example, weight at maturation is given by, *W*_*mat*_ = η_*m*_ *W*_*inf*_, where the default value of η_*m*_ is 0·25.

In the multispecies model, individual species are resolved in detail and each has distinct life history, feeding and reproduction parameters.

In the trait-based and multispecies models, egg production and recruitment are dependent on the amount of food consumed. The default recruitment function is based on a ‘Beverton–Holt’ type function (Beverton & Holt 1957) where the recruitment flux approaches a maximum as egg production increases. Users are able to specify alternative recruitment functions for each species.

In all three model versions, the species are defined by a series of parameters (Table S1), some of which have default values. Each model version also uses a range of species-independent parameters (Table S2). More detail on the differences and example uses of each of these approaches is provided in Section 4 of the vignette.

## Using mizer

Although size spectrum models can be complex, *mizer* has been developed so that setting up a model, running a simulation and inspecting the results are straightforward.

A class, *MizerParams*, is used for model parameters. For community and trait-based models, wrapper functions *set_community_model()* and *set_trait_model()* can be used to easily create an appropriate *MizerParams* object. All function arguments have default values to allow the user to quickly set up and explore the dynamics of size spectrum models. However, these default values may not be appropriate for all case studies. To set up the multispecies model, the *MizerParams()* constructor method is used, which has two main arguments: a data frame of species parameters (Table S1) and an interaction matrix which holds the species prey preference. A demonstration multispecies data set is included in the package, based on the North Sea (Blanchard *et al*. 2014).

Simulations are run by calling *project()*. Fishing mortality is controlled by the fishing effort of each gear and can be specified independently by gear through time, to explore the impact of changing fishing pressures on different groups of species. Additionally, it is possible to use observed historical fishing pressures in the simulations (see the vignette and Blanchard *et al*. 2014).

The output of *project()* is an object of class *MizerSim* which contains the simulation results including species abundances by size and time. The results can be explored using plot, summary and indicator methods (Table S3). For example, one indicator of the impact of fishing is the slope of the community spectrum. This can be calculated using *getCommunitySlope()*. The size range of the calculation can be specified to capture the size range that is sampled by field surveys.

## Examples

Two *mizer* examples exploring fishing impacts on different parts of the community are shown below. The code is provided in the additional supporting material (Appendix S1). Further examples can be found in the package vignette.

**Figure 2:**
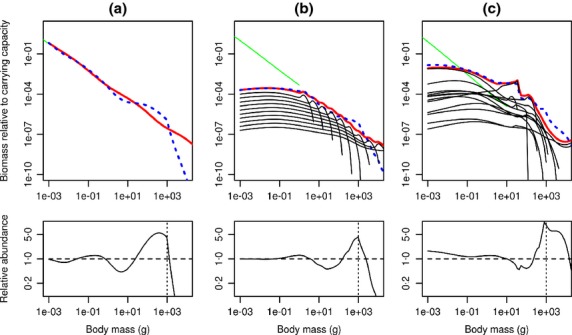
Comparing unfished and fished communities with the three model versions: (a) community, (b) trait-based and (c) multispecies based on the North Sea. Top row: biomass of the background resource (green), total community biomass (red) and individual species biomasses (black) in the unfished case; total community biomass in the fished case (blue dashed), all relative to the carrying capacity of the background resource at 10^−3^ g. In the trait-based model, the asymptotic weights of the 10 species in the model are evenly spread over a log scale, giving the parallel species biomass lines seen. In the multispecies model, the asymptotic weights are species-dependent and hence the species biomass lines are not parallel. Bottom row: relative total abundance of fished and unfished communities. Fishing is with knife-edge selectivity at 1000 g (vertical dashed line).

The first example simulates a trophic cascade using all three models (Fig.2). Here all species are fished with a knife-edge selectivity so that only individuals >1000 g are selected (a simplification as selectivity is normally species-specific, possible with *mizer*). The simulations are run to equilibrium to investigate equilibrium fished and unfished states (details can be found in the vignette). The fishing pressure lowers the relative abundance of fish >1000 g and reduces predation pressure on smaller prey, leading to an increase in their abundance. This in turn increases predation mortality on even smaller sizes, reducing their abundance and so on, causing a size-structured cascade.

**Figure 3:**
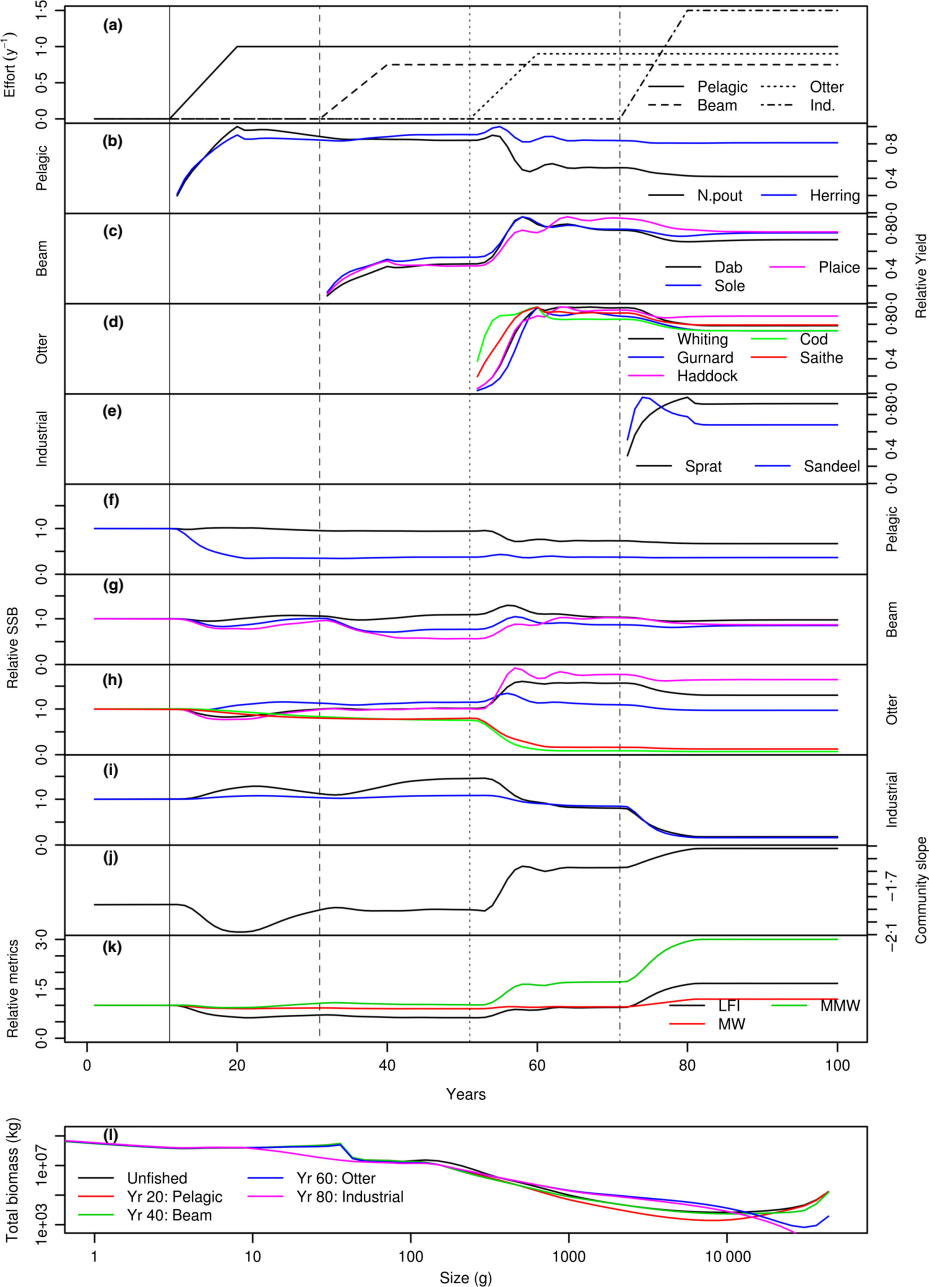
Response of the North Sea multispecies model, fished by four gears (pelagic, beam, otter and industrial) with the gears ‘turned on’ in sequence through time. Each gear selects a different group of species. (a) fishing effort of each gear per year. (b–e) species-specific yield, relative to the maximum yield of each gear. Only the species caught by that gear are shown in each panel. (f–i) Spawning stock biomass (SSB) relative to the unfished SSB of the species caught by that gear. (j) Slope of the community abundance. (k) Large fish indicator (LFI), mean weight (MW) and mean maximum weight (MMW) relative to their unfished levels. Indicators in (j) and (k) are based on individuals between 10 and 5000 g. The vertical lines through (a–k) show the timings of when each gear is switched on. (l) Total community biomass spectra at the start and at the times when each fishing gear reaches its maximum effort through the simulation.

In the second example, a multispecies model based on the North Sea is fished by four different gears (pelagic, otter, beam and industrial), each of which selects a different group of the 12 species in the model (Fig.3). The gears have knife-edge selectivity, but the size at selection differs between gears (again, this is a simplification, as selectivity is usually gear and species-specific). The simulation is run for 100 years, from equilibrium unfished abundances. The large predatory species are cod, whiting, saithe and haddock. Sprat, sandeel and herring are smaller forage fish that consume the resource spectrum. The fishing gears are ‘turned on’ in sequence so that fishing pressure on different parts of the community varies with time. The community-wide impacts of fishing are manifest in the abundances, yields and community metrics. For example, when the otter gear starts fishing, the beam gear yields increase as the predation pressure from species caught by the otter gear declines. The slope of the community also becomes less negative. Turning on the industrial fishery targeting forage fish leads to a marked increase in the mean maximum weight in the community and has a negative impact on the yields of the species caught by the other gears.

## Conclusions and future directions

The *mizer* package can be used by fisheries scientists and ecologists who wish to use size spectrum models to explore the impacts of exploitation on aquatic communities. The usefulness of community indicators, such as the slope of the community spectrum, can also be tested by evaluating how responsive they are to changes in fishing pressure. Additionally, the package may be of use for teaching the principles of size spectrum modelling.

Several developments have been planned for future releases. Currently, the model is purely deterministic, and it is not possible to quantify the uncertainty in the results. The inclusion of stochasticity, for example in the recruitment function, will help to address this.

For the model to simulate a real ecosystem, it must be parameterized. This is done outside *mizer* based on either the literature or a statistical analysis of growth, maturation and diet data (Blanchard *et al*. 2014). Calibration of the least-known parameters (e.g. carrying capacity of the background resource and species maximum recruitment) is required. Statistical parameter estimation to fit the emergent abundance patterns of dynamic size spectrum models to data is an area of current and future methodological development and could be carried out through the use of optimization (Blanchard *et al*. 2014), maximum likelihood or Bayesian inference algorithms.

## Data accessibility

This paper does not use data.

## Supporting Information

Table S1 Column names and default values for the species parameters in the species data frame used when making a *MizerParams* object. **Table S2.** Species-independent model parameters and the default values for the three model versions. **Table S3.** Summary, indicator and plotting methods for the *MizerSim* class to explore the results after running a simulation.

Appendix S1 R code for examples.
