# Supplementary material for: mizer: an R package for multispecies, trait-based and community size spectrum ecological modelling
Source: Methods Ecol Evol. 2014 Sep 23;5(10):1121–5. doi: 10.1111/2041-210X.12256 (PMC4384942; doi:10.1111/2041-210X.12256)
Supplement: Table S1 — Column names and default values for the species parameters in the species data frame used when making a MizerParams object. Table S2. Species-independent model parameters and the default values for the three model versions. Table S3. Summary, indicator and plotting methods for the MizerSim class to explore the results after running a simulation. [file mee30005-1121-sd1.docx]

Supporting material for a draft submission for an Applications article in Methods in Ecology and Evolution

**mizer: an R package for multispecies, trait-based and community size spectrum ecological modelling**

Finlay Scott, Julia L. Blanchard and Ken H. Andersen

TABLE S1

Column names and default values for the species parameters in the species data frame used when making a *MizerParams* object. Each row contains the parameters for a species. The same structure is used for all three model versions, but the default values may be different. When creating a multispecies model the data frame needs to be made by hand and passed to the *MizerParams()* constructor method. Due to the default values it is possible to make a multispecies model by only supplying the *species*, *w_inf*, *w_mat*, *beta*, *sigma*, *k_vb* and *r_max* columns. When making community and trait-based models the data frame is generated internally by the wrapper functions *set_community_model()* and *set_trait_model()* and does not need to be created by the user. The unit of time of the model is generally taken to be year. However, other units may be used so long as it is consistent across parameters.

| Column name | Description | Units | Community model | Trait-based model | Multispecies model |
| --- | --- | --- | --- | --- | --- |
| species | Species name |  | *Community* | 1 to i | Species dependent |
| w_inf | Asymptotic size | g | NA | Evenly spread on log scale over size spectrum | Species dependent |
| w_mat | Size at maturation | g | NA | 0.25 *w_inf* | Species dependent |
| beta | Preferred PPMR |  | 100 | 100 | Species dependent |
| sigma | Width of the weight selection function |  | 2.0 | 1.3 | Species dependent |
| k_vb | von Bertalanffy *K* parameter | year^-1^ | NA | NA | Only used to estimate *h* if not provided |
| h | Maximum food intake rate | g^1-n^/year | 10 | 30 | Estimated from *k_vb* if not provided |
| gamma | Volumetric search rate |  | Estimated from *h* if not provided | Estimated from *h* if not provided | Estimated from *h* if not provided |
| ks | Standard metabolism coefficient | g^1-p^/year | 0 | 4 | 0.2 *h* |
| z0 | Background mortality constant | g^1-n^/ year | 0.1 | 0.6 *w_inf* ^-1/3^ | 0.6 *w_inf* ^-1/3^ |
| k | Activity coefficient |  | 0 | 0 | 0 |
| alpha | Assimilation efficiency |  | 0.2 | 0.6 | 0.6 |
| erepro | Reproductive efficiency |  | NA | 1 | 1 |
| gear | Name of fishing gear that selects that species |  | *species* | *species* | *species* |
| sel_func | Name of selectivity function that determines the selectivity curve |  | *knife_edge* | *knife_edge* | *knife_edge* |
| catchability | Catchability of fishing gear on that species |  | 1 | 1 | 1 |
| other selectivity columns | Parameters used by selectivity function |  | *knife_edge_size* = 1000 | *knife_edge_size* = 1000 | *knife_edge_size* = 1000 |
| r_max | Max. recruitment flux | g/year | NA | The maximum recruitment of an unexploited system, calculated using the theory of a size spectrum at equilibrium assuming that the feeding level is constant (Andersen and Pedersen, 2010) | Species dependent |

TABLE S2

Species-independent model parameters and the default values for the three model versions.

| Parameter | Description | Units | Community model | Trait-based model | Multispecies model |
| --- | --- | --- | --- | --- | --- |
| min_w | Smallest size of the community spectrum | g | 1e-3 | 1e-3 | 1e-3 |
| max_w | Largest size of the community spectrum | g | 1e6 | 1.1e5 | max(w_inf)*1.1 |
| no_w | Number of size bins in the community spectrum |  | 100 | 100 | 100 |
| min_w_pp | Smallest size of the background resource spectrum | g | 1e-10 | 1e-10 | 1e-10 |
| no_w_pp | Number of extra size bins of the background resource spectrum (total number of size bins is no_w_pp + no_w) |  | 30 | 30 | 30 |
| w_pp_cutoff | Cut off size of the background resource spectrum | g | 1e-3 | 1 | 10 |
| n | Scaling of intake |  | 2/3 | 2/3 | 2/3 |
| p | Scaling of the standard metabolism |  | NA | 0.75 | 0.7 |
| q | Search volume exponent |  | 0.8 | 0.9 | 0.8 |
| r_pp | Growth rate of the background resource spectrum | g^1-p^ / year | 10 | 4 | 10 |
| kappa | Carrying capacity of the background resource spectrum | g^λ-1^ | 1000 | 0.005 | 1e11 |
| lambda | Exponent of the background resource spectrum |  | 2 + q - n | 2 + q – n | 2 + q – n |
| f0 | Feeding level of small individuals feeding mainly on the background resource. Used to calculate *h* and *gamma* if not provided in the species data frame (see Table S2) |  | 0.7 | 0.5 | 0.6 |

TABLE S3

Summary, indicator and plotting methods for the *MizerSim* class to explore the results after running a simulation. All methods take an object of class *MizerSim*. The indicator methods may take some additional arguments, for example to specify ranges over which to calculate the indicator. Additional arguments are noted below. More details of these methods and their usage are available in the package vignette on CRAN and in the package documentation.

| Summary methods | | |
| --- | --- | --- |
| Method | Returns | Description |
| getSSB | Two dimensional array (time x species) | Total Spawning Stock Biomass by species through time calculated as the sum of the mass of all mature individuals (g). |
| getBiomass | Two dimensional array (time x species) | Total biomass by species through time (g). |
| getN | Two dimensional array (time x species) | Total abundance by species through time. |
| getFeedingLevel | Three dimensional array (time x species x size) | Feeding level by species by size through time. Feeding level is the amount of food consumed by a predator based on food availability, search volume and maximum intake. It has a value from 0 to 1 (fully satiated) and is used to calculate predation rate. |
| getM2 | Three dimensional array (time x species x size) | The predation mortality imposed on each species by size through time (year^-1^). |
| getFMort | Three dimensional array (time x species x size) | Total fishing mortality on each species by size through time (year^-1^). |
| getFMortGear | Four dimensional array (time x gear x species x size) | Fishing mortality on each species by gear and size through time (year^-1^). |
|  |  |  |
| getYieldGear | Three dimensional array (time x species x size) | Total yield by species and gear through time (g/year). |
| getYield | Two dimensional array (time x species) | Total yield by species across all gears through time (g/year). Effectively sums the ouput of *getYieldGear* over the fishing gears. |
| getPredRate | Three dimensional array (predator species x predator size x prey size) | Predation rate of each predator at size on prey size (year^-1^). |
| Indicator methods | | |
| Method | Returns | Description |
| getProportionOfLargeFish | A vector with values at each time step. | Proportion of large individuals in the community through time. The proportion is based on either length or weight (through the *biomass_proportion* argument ). The threshold value (i.e. what is a ‘large’ fish) can also be specified. See the help file for more details. |
| getMeanWeight | A vector with values at each time step. | Mean weight of the community through time (g). Calculated as the total biomass of the community divided by total abundance. The size range over which to calculate the values can be specified. |
| getMeanMaxWeight | Depends on the *measure* argument. If measure = “both" you get a matrix with two columns, one with values by numbers, the other with values by biomass at each time step. If measure = ”numbers" or “biomass" you get a vector of the respective values at each time step. | Mean maximum weight of the community through time calculated as the sum of the w_inf * abundance of each species, divided by the total community abundance. Abundance iscalculated by numbers or biomass (g), specified through an additional *measure* argument. The size range over which to calculate the values can be specified. |
| getCommunitySlope | A data.frame with four columns: time step, slope, intercept and R^2^ value. | Calculates the slope and other measures of the community abundance spectrum through time by performing a  linear regression on the logged total numerical abundance or biomass and logged body size. The size range over which to calculate the values can be specified. |
| Plotting methods  (for the Community model only one ‘species’ is present so only one line will be drawn for some of the plots) | | |
| plotBiomass | Total biomass by species through time. | |
| plotSpectra | Abundance (biomass or numbers) spectra of each species and the background community | |
| plotFeedingLevel | Feeding level of each species against size. | |
| plotM2 | Predation mortality of each species against size. | |
| plotFMort | Total fishing mortality of each species against size. | |
| plotYield | Total yield of each species across all fishing gears against time. | |
| plotYieldGear | Total yield of each species by gear against time. | |
| plot | Summary plot that produces 5 plots (*plotFeedingLevel*, *plotBiomass*, *plotM2*, *plotFMort* and *plotSpectra*). | |
